# Development of an adjuvanted nanoparticle vaccine against influenza virus, an *in vitro* study

**DOI:** 10.1371/journal.pone.0237218

**Published:** 2020-08-06

**Authors:** Kamonthip Rungrojcharoenkit, Panya Sunintaboon, Damon Ellison, Louis Macareo, Panuwat Midoeng, Preamrudee Chaisuwirat, Stefan Fernandez, Sukathida Ubol

**Affiliations:** 1 Department of Microbiology, Faculty of Science, Mahidol University, Bangkok, Thailand; 2 Department of Virology, Armed Forces Research Institute of Medical Sciences, Bangkok, Thailand; 3 Department of Chemistry, Faculty of Science, Mahidol University, Bangkok, Thailand; 4 Division of Pathology, Army Institute of Pathology, Phramongkutklao Hospital, Bangkok, Thailand; University of South Dakota, UNITED STATES

## Abstract

Influenza is an infectious respiratory illness caused by influenza viruses. Despite yearly updates, the efficacy of influenza vaccines is significantly curtailed by the virus antigenic drift and antigenic shift. These constant changes to the influenza virus make-up also challenge the development of a universal flu vaccine, which requires conserved antigenic regions shared by influenza viruses of different subtypes. We propose that it is possible to bypass these challenges by the development of an influenza vaccine based on conserved proteins delivered in an adjuvanted nanoparticle system. In this study, we generated influenza nanoparticle constructs using trimethyl chitosan nanoparticles (TMC nPs) as the carrier of recombinant influenza hemagglutinin subunit 2 (HA2) and nucleoprotein (NP). The purified HA2 and NP recombinant proteins were encapsulated into TMC nPs to form HA2-TMC nPs and NP-TMC nPs, respectively. Primary human intranasal epithelium cells (HNEpCs) were used as an *in vitro* model to measure immunity responses. HA2-TMC nPs, NP-TMC nPs, and HA2-NP-TMC nPs (influenza nanoparticle constructs) showed no toxicity in HNEpCs. The loading efficiency of HA2 and NP into the TMC nPs was 97.9% and 98.5%, respectively. HA2-TMC nPs and NP-TMC nPs more efficiently delivered HA2 and NP proteins to HNEpCs than soluble HA2 and NP proteins alone. The induction of various cytokines and chemokines was more evident in influenza nanoparticle construct-treated HNEpCs than in soluble protein-treated HNEpCs. In addition, soluble factors secreted by influenza nanoparticle construct-treated HNEpCs significantly induced MoDCs maturation markers (CD80, CD83, CD86 and HLA-DR), as compared to soluble factors secreted by protein-treated HNEpCs. HNEpCs treated with the influenza nanoparticle constructs significantly reduced influenza virus replication in an *in vitro* challenge assay. The results indicate that TMC nPs can be used as influenza vaccine adjuvants and carriers capable of delivering HA2 and NP proteins to HNEpCs.

## Introduction

Influenza is an infectious respiratory illness caused by influenza viruses. Annually, influenza causes between 3–5 million cases of severe illness and between 290,000 to 650,000 deaths [1]. Globally, the control of influenza infections in humans includes influenza vaccination and antiviral drugs, which target the viral neuraminidase (NA) or the matrix-2 (M2) proteins. However, the efficacy of antivirals is limited by emerging resistance to both M2 and NA inhibitors [2–4].

Currently, most common commercial influenza vaccines are administered by intramuscular or subcutaneous routes which induce neutralizing serum IgG antibodies. However, these vaccines are poor stimulators of secretory IgA antibodies at the respiratory mucosa [5, 6]. Intranasal immunization can induce both systemic and mucosal immune responses [7–9]. However, intranasal vaccines available in the market are live-attenuated and are unsuitable for administration to young children, the elderly or immune-compromised patients due to safety concerns [10]. Vaccine adjuvants such as alum and MF59 are used to enhance immune response to recombinant, subunit and killed vaccines by potentiating and prolonging the immune responses to antigens, and by reducing the amount of antigen required and the frequency of booster immunizations [11]. Adjuvants, like MF59 (oil-in-water emulsion), can also improve immune responses in the elderly and in immunocompromised patients. However, as demonstrated by MF59 in influenza vaccines, it may occasionally fail to increase antibody titers in healthy individuals and raise widespread concerns about safety due to local and systemic adverse reactions [12, 13].

Recent research indicates that nanoparticles can be used as both potent adjuvants and vaccine delivery systems. Nanoparticles are biodegradable and biocompatible. They have low toxicity and protect antigens or DNA from damage [14–16]. Chitosan is a natural cationic polysaccharide consisting of N-acetylglucosamine and D-glucosamine units which are obtained by deacetylation of chitin. In recent years, vaccine carriers based on chitosan and its derivatives became popular for delivery of proteins via mucosal routes [17]. Chitosan absorbs protein/antigen and efficiently adhere to epithelial cells transporting bioactive molecules towards M cells [18, 19]. Chitosan is biocompatible and of low toxicity. Chitosan is a cationic compound, thus, it could interact with negatively charged mucin by electrostatic interactions. This interaction increases retention time at the mucosal site [18–20]. However, chitosan is limited by poor solubility at physiological pH [21, 22]. In contrast, a derivative of chitosan, trimethyl chitosan (TMC), exhibits great solubility in aqueous solution at neutral pH. These attributes make TMC an attractive alternative to chitosan for the design of protein-loaded nanoparticles vaccines. Several studies have shown that TMC nanoparticles (TMC nPs) exert adjuvant-like effects on dendritic cells and can be used as potent adjuvants and delivery systems capable of inducing mucosal immunity [23, 24]. Moreover, TMC nPs are found to display mucoadhesive properties in their prolongation of residence time at the absorption sites [25]. TMC nPs loaded with influenza subunit antigens or whole inactivated influenza viruses increased immune responses and the efficiency of intranasal administered vaccines in mice [23, 26].

Influenza vaccines require yearly updates as their efficacy diminishes due to antigenic drift and/or antigenic shift. Development of a universal influenza vaccine targets conserved regions in different influenza subtypes. Important among these regions is the glycoprotein hemagglutinin (HA), found in the surface envelope of influenza viruses and consisting of two subunits, HA1 and HA2 [27]. HA plays roles in virus entry and infectivity. HA2 is highly conserved [28] and stimulates protective antibodies against specific strains and cross-protective antibodies against several subtypes [29–33]. Nucleoprotein (NP), a structural protein which participates in encapsidation of the negative strand viral RNA, is also highly conserved [34–37]. NP is immunogenic, inducing antigen specific and crossmatching cytotoxic T lymphocytes [38–40]. *In vivo* models revealed that several administrations of a recombinant NP antigen presented in various vaccine platforms induced protective immunity [41–46]. Given their highly conserved regions, a universal influenza vaccine composed of HA2 and NP should provide protection against genetic drift [47–49]. Unfortunately, HA2- and NP-based vaccines are of low immunogenicity. Addition of an adjuvant and carrier system for intranasal administration may improve the immunogenicity of HA2- and NP- based vaccines.

Here, we generated influenza nanoparticle constructs by using TMC nPs as an adjuvant containing HA2 and NP proteins. We investigated the effects of these influenza nanoparticle constructs *in vitro* using primary human intranasal epithelium cells (HNEpCs) and human monocyte-derived dendritic cells (MoDCs).

## Materials and methods

### Cells and viruses

Madin-Darby canine kidney cells (MDCK) (CCL34, ATCC) were cultured in growth medium (D-MEM (Gibco) containing 10% heat inactivated fetal bovine serum (HIFBS, Gibco), 100 U/ml penicillin and 100 μg/ml streptomycin) and incubated at 37°C and 5% CO_2_.

Primary human nasal epithelial cells (HNEpCs) (C-12620, PromoCell, Germany), were grown in airway epithelial cell growth medium (PromoCell) at 37°C and 5% CO_2_. Cells were grown in tissue culture flasks coated with purified collagen (50 μg/ml) (Advanced BioMatrix, USA).

A/California/07/2009 (H1N1) was used to prepare HA2 and NP proteins and used for *in vitro* challenge. A/California/07/2009 was propagated in MDCK cells in maintenance medium (D-MEM containing 0.2% bovine serum albumin (BSA), 100 U/ml penicillin and 100 μg/ml streptomycin with TPCK trypsin at a 2 μg/ml). Cells were further incubated at 37°C and 5% CO_2_ for 5 days. The stock viruses were centrifuged at 2,000 rpm for 10 min at 4°C to remove the cell debris prior to aliquoted and stored at -80°C.

### Preparation of HA2 and NP proteins

Influenza genomic RNAs was extracted from A/California/07/2009 using QIAamp viral RNA kit (QIAGEN) and reverse transcribed into cDNA by SuperScript® III First-Strand Synthesis System (Invitrogen, USA) using random primers, according to the manufacturer’s protocol. To generate HA2 and NP gene fragments, cDNA products were amplified by polymerase chain reaction (PCR) (Toyobo Life Science, Japan) using primer pairs as follows; For HA2 cDNA fragment (residues 1–185), forward primer 5'- CTG AAT TCT CGG CCT ATT TGG GGC CAT T -3' and reverse primer 5'- GGT CTA GAG CCT GGT AAA TCC TTG TTG ATT C -3'. For NP cDNA fragment (residues 1–499), forward primer 5'- TTG AAT TCT CAT GGC GTC TCA AGG CAC C -3' and reverse primer 5'- CAT CTA GAG CAC TGT CAT ACT CCT CTG C -3'. The amplified PCR products were purified and cloned into pGEM-T cloning vectors (Promega, USA) according to the manufacturer’s protocol. Purified pGEM-T HA2 and pGEM-T NP plasmids were sub-cloned into plasmid pPICZαB (Invitrogen) before being electroporated into *Pichia pastoris*. The integration of HA2 and NP genes in the transformed clones was confirmed using manufacturer’s protocol.

To obtain the HA2 and NP proteins, the expression of recombinant HA2 and NP proteins was induced with 1% methanol in culture medium for 96 h. For HA2 proteins, the cells were harvested and broken by glass beads. The supernatant was collected. For secreted NP proteins, the culture medium of the methanol-induced yeast cells was collected and concentrated by tangential flow filtration. Solubilized HA2 and secreted NP proteins were purified by His-tag affinity chromatography (ProBond, Invitrogen). The purified HA2 and NP proteins were confirmed by immunoblotting using mouse anti-histidine monoclonal antibodies (Invitrogen), mouse monoclonal antibodies against influenza HA (IT-003-SW, Immune Technology Corp, USA) or rabbit polyclonal antibodies against influenza NP (clone C43, ab128193, Abcam, UK). The membranes were further incubated with horseradish peroxidase conjugated with rabbit anti-mouse IgG (KPL, USA) or goat anti-rabbit IgG (KPL) against the mouse and rabbit antibodies, respectively. The reactive protein bands were then visualized with SuperSignal West Pico chemiluminescence substrate (Pierce Biotechnology, USA). The levels of endotoxin contaminants in purified HA2 and NP were tested using the Limulus Amebocyte lysate assay (QCL-1000; Pierce, Rockford, USA) and found to be <0.1 EU/mg.

### Formulation and characterization of trimethyl chitosan nanoparticles-based HA2 and NP

Empty TMC nPs, HA2-TMC nPs and NP-TMC nPs were prepared using ionic gelation. Briefly, empty TMC nPs were prepared by drop-wise addition of sodium tripolyphosphate (TPP) solution (0.265 mg/ml) into the TMC solution (2 mg/ml) containing 1% (w/w) Tween 80 in HEPES buffer for 1 h. For HA2-TMC nPs and NP-TMC nPs, TPP solution was mixed with HA2 or NP (0.3 mg/ml) and then drop-wised into the TMC solution as above. Empty TMC nPs, HA2-TMC nPs and NP-TMC nPs suspensions were centrifuged at 10,000 x g for 10 min at 4°C on a 10 μl glycerol bed. The supernatants (free protein) were then collected to measure loading efficiency and the pellets were resuspended in 5 mM HEPES buffer, pH 7.4, to measure size, nanoparticle size distribution and surface charge as zeta-potential of nanoparticles using a Zetasizer (NanoZS 4700, Malvern Instruments, UK). The nanoparticle size distribution was reported as a polydispersity index (PDI), ranging from 0 for an entirely monodisperse suspension to 1 for a completely heterodisperse system. The amounts of protein entrapped in the TMC nPs were determined by microbicinchoninic acid protein assay (μBCA, Pierce) using BSA as a standard. Loading efficiency for HA2 and NP was calculated as follows:

$$Loading\;efficiency=\frac{\left(Total\;amount\;of\;protein-free\;protein\right)}{Total\;amount\;of\;protein}\times 100\%$$

To elucidate whether encapsulation of TMC nPs on HA2 and NP proteins altered their antigenicity, HA2-TMC nPs and NP-TMC nPs were destabilized by adding 10% (w/v) NaCl solution to 4.5 ml of the nanoparticles. The released proteins were tested by SDS-PAGE and visualized by staining with Coomassie blue R250. Immunoblotting analysis was performed using monoclonal antibodies against HA (Immune Technology Corp) or rabbit polyclonal antibodies against NP (Abcam).

### Cytotoxicity assay

HNEpCs were seeded on 12-well plate (1 x 10^5^ cells/well) and cultured for 24 h at 37°C in a 5% CO_2_ incubator. HNEpCs monolayers were washed with phosphate buffered saline (PBS) and then treated with protein alone (HA2, NP or mixture of HA2 and NP (HA2+NP) at 15 μg/ml), empty TMC nPs, HA2-TMC nPs, NP-TMC nPs, mixture of HA2-TMC nPs and NP-TMC nPs (HA2-NP-TMC nPs) at 25 and 100 μg/ml or lipopolysaccharide (LPS) at 1 μg/ml. Cell viability was evaluated by trypan blue staining at 24 and 48 h.

### Cellular uptake of HA2-TMC nPs and NP-TMC nPs

To investigate the cellular uptake of HA2-TMC nPs and NP-TMC nPs, HNEpCs were treated with protein (HA2 or NP at 15 μg/ml), empty TMC nPs (100 μg/ml), or with various concentrations of HA2-TMC nPs or NP-TMC nPs (25 and 100 μg/ml) at 37°C and 5% CO_2_. Cells were collected after 24 and 48 h of treatment. Cells were washed with Perm wash (BD Biosciences, USA) and permeabilized with Cytofix/Cytoperm (BD Biosciences). Intracellular HA2 was stained with mouse anti-histidine monoclonal antibodies (Invitrogen). PE-conjugated goat anti-mouse (BD Biosciences) was used as secondary antibodies. Intracellular NP was stained with mouse anti-influenza NP antibodies conjugated FITC (clone A1, MAB8257F, Millipore, USA). Mean fluorescence intensity (MFI) and frequency of fluorescence positive cells were measured by BD LSR Fortessa Cell Analyzer (BD Biosciences).

### Cytokine and chemokine productions

HNEpCs were treated at 37°C and 5% CO_2_ as follows: negative control (medium), positive control (LPS at 1 μg/ml), protein alone (HA2, NP or HA2+NP at 15 μg/ml), empty TMC nPs, HA2-TMC nPs, NP-TMC nPs or HA2-NP-TMC nPs at 25 and 100 μg/ml. Supernatants were collected at 24 and 48 h and tested individually for cytokine and chemokine productions using the Bio-Plex human cytokine assay kit (Bio-Rad Laboratories, USA) following the manufacturer protocol. Amounts of IL-6, IL-1β, TNF-α, G-CSF, GM-CSF, IL-7, MCP-1, MIP-1β, IL-8, IL-2, IL-12p70, IL-17, IFN-γ, IL-4, IL-5, IL-10, IL-13 were quantitated simultaneously. The concentration of IFN-α was measured separately using an ELISA kit (VeriKine™, USA).

### Isolation of human monocyte-derived dendritic cells (MoDCs)

Peripheral blood mononuclear cells (PBMC) were isolated from buffy coats of healthy blood donors that were obtained from a blood bank service at Phramongkutklao hospital. CD14^+^ monocyte cells were purified by CD14 magnetic microbeads by MACs (Miltenyi Biotech, Germany) according to manufacturer’s instruction. To generate immature dendritic cells (iDCs), purified monocytes were cultured in RPMI-1640 medium containing 10% HIFBS, 100 ng/ml of IL-4 (Miltenyi Biotech) and 100 ng/ml of GM-CSF (Miltenyi Biotech) at 37°C and 5% CO_2_. The complete medium was changed every other day for 7 days. The numbers of iDCs were measured by goat anti-human CD11c-FITC conjugated (BD Pharmingen).

### The effect of soluble factors secreted by influenza nanoparticle construct-treated HNEpCs on MoDCs maturation

To determine the effect of soluble factors secreted by influenza nanoparticle construct-treated HNEpCs on MoDCs maturation, HNEpCs were treated as follows: medium, protein alone (HA2, NP or HA2+NP at 15 μg/ml), empty TMC nPs, HA2-TMC nPs, NP-TMC nPs and HA2-NP-TMC nPs at 100 μg/ml or 1 μg/ml of LPS as positive control. The supernatants of HNEpCs were collected at 48 h and added into MoDCs. MoDCs were cultured in 24 well plate and collected at 24 and 48 h to measure the expression of DCs maturation markers (CD80, CD83, CD86 and HLA-DR, (BD Pharmingen)) by BD LSR Fortessa Cell Analyzer (BD Biosciences).

### *In vitro* challenge for detection of antiviral activity

HNEpCs were seeded on 12-well plates (1 x 10^5^ cells/well) and cultured for 24 h at 37°C and 5% CO_2_. HNEpCs were treated as follows: protein alone (HA2, NP or HA2+NP at 15 μg/ml), empty TMC nPs, HA2-TMC nPs, NP-TMC nPs and HA2-NP-TMC nPs at 100 μg/ml and medium as control. The treatments were carried out at 37°C and 5% CO_2_ for 24 h. HNEpCs supernatants were collected and kept at 37°C and 5% CO_2_ for later use. Treated-HNEpCs were washed twice with PBS and then inoculated with A/California/07/2009 (H1N1) at an MOI of 1.5 PFU/cell at 37°C and 5% CO_2_ for 1.5 h. After incubation, cells were washed twice with PBS, and the spent supernatants were returned to the HNEpCs. The supernatants were again collected 24 and 72 h later and the secreted influenza viruses in the supernatant were quantified as tissue culture infectious doses (TCID_50_). Medium-treated HNEpCs infected with A/California/07/2009 was used as control.

### Tissue culture infectious dose (TCID_50_)

This assay was modified from a previously reported procedure [50]. Briefly, cell supernatants containing A/California/07/2009 (H1N1) were diluted in virus diluent (D-MEM supplemented with 1% BSA, 100 U/ml penicillin, 100 μg/ml streptomycin and 20 mM HEPES) in 96-well plates. Virus was diluted in ½ log_10_ dilutions. 100 μl of MDCK cells (1.5 x 10^5^ cells/ml) were added to each well of the microtiter plates and allowed to adsorb at 37°C and 5% CO_2_ for 18 to 20 h. After incubation, the media were removed and cells were washed three times with PBS. Cells were fixed with cold 80% acetone in PBS at room temperature (RT) for 10 min. Cells were then washed five times with PBS containing 0.05% Tween 20 (PBST), followed by the addition of mouse monoclonal antibody to the influenza A nucleoprotein (Millipore) at RT for 1 h. The plates were washed five times with PBST and incubated with horseradish peroxidase-labeled goat anti-mouse IgG antibody at RT for 1 h. Finally, cells were then washed five times with PBST, and 100 μl/well of Sureblue TMB solution (KPL, Inc., USA), HRP substrate was added and incubated in the dark for 30 min. The reaction was stopped with 0.5 M sulfuric acid. Absorbance was determined at 450 nm with microplate reader. The TCID_50_ titer was calculated by the Reed and Muench method [51].

### Statistical analysis

Data were analyzed using GraphPad Prism version 7.0 for windows (GraphPad Software). All data was reported as mean ± standard deviation (SD) values for all measurements. Statistical analysis was performed using student’s *t*-test or one-way ANOVA with Tukey's post-test when appropriate *P* values less than 0.05 were considered statistically significant.

### Ethical considerations

Ethical approval for this study were reviewed by Mahidol university central institutional review board (MU-CIRB 2018/019.2704) and WRAIR IRB/HSPB (WRAIR Policy 12–09 section 8.a) and declared exemption from review.

## Results

### Characterization of HA2-TMC nPs and NP-TMC nPs

To generate HA2-TMC nPs and NP-TMC nPs, HA2 and NP proteins were encapsulated into TMC nPs by ionic gelation. As shown in Table 1 and S1 Fig, the diameter of empty TMC nPs was 282.2 ± 6.6 nm, with a narrow size distribution (0.254 ± 0.016) and positive surface charge (+14.6 ± 1.0 mV). HA2-TMC nPs had a diameter of 422.9 ± 12.2 nm with positive surface charge (11.4 ± 0.2 mV). The size distribution of HA2-TMC nPs was 0.388 ± 0.008, as shown by polydispersity index (PDI). NP-TMC nPs had an average diameter of 489.8 ± 11.7 nm with a positive surface charge (11.6 ± 0.2 mV) and PDI of 0.424 ± 0.012. Loading efficiency of HA2 and NP into TMC nPs was 97.9 ± 0.1% and 98.5 ± 0.1%, respectively. The amounts of encapsulated HA2 or NP protein into TMC nPs (HA2-TMC nPs and NP-TMC nPs) at 25 and 100 μg/ml were 3.75 and 15 μg, respectively.

**Table 1:** Nanoparticles properties.

Nanoparticles	Particle size (nm)	Polydispersity index (PDI)	Zeta-potential (mV)	Loading efficiency (%)
empty TMC nPs	282.2 ± 6.6	0.254 ± 0.016	14.6 ± 1.0	-
HA2-TMC nPs	422.9 ± 12.2	0.388 ± 0.008	11.4 ± 0.2	97.9 ± 0.1
NP-TMC nPs	489.8 ± 11.7	0.424 ± 0.012	11.6 ± 0.2	98.5 ± 0.1

**Fig 1:**
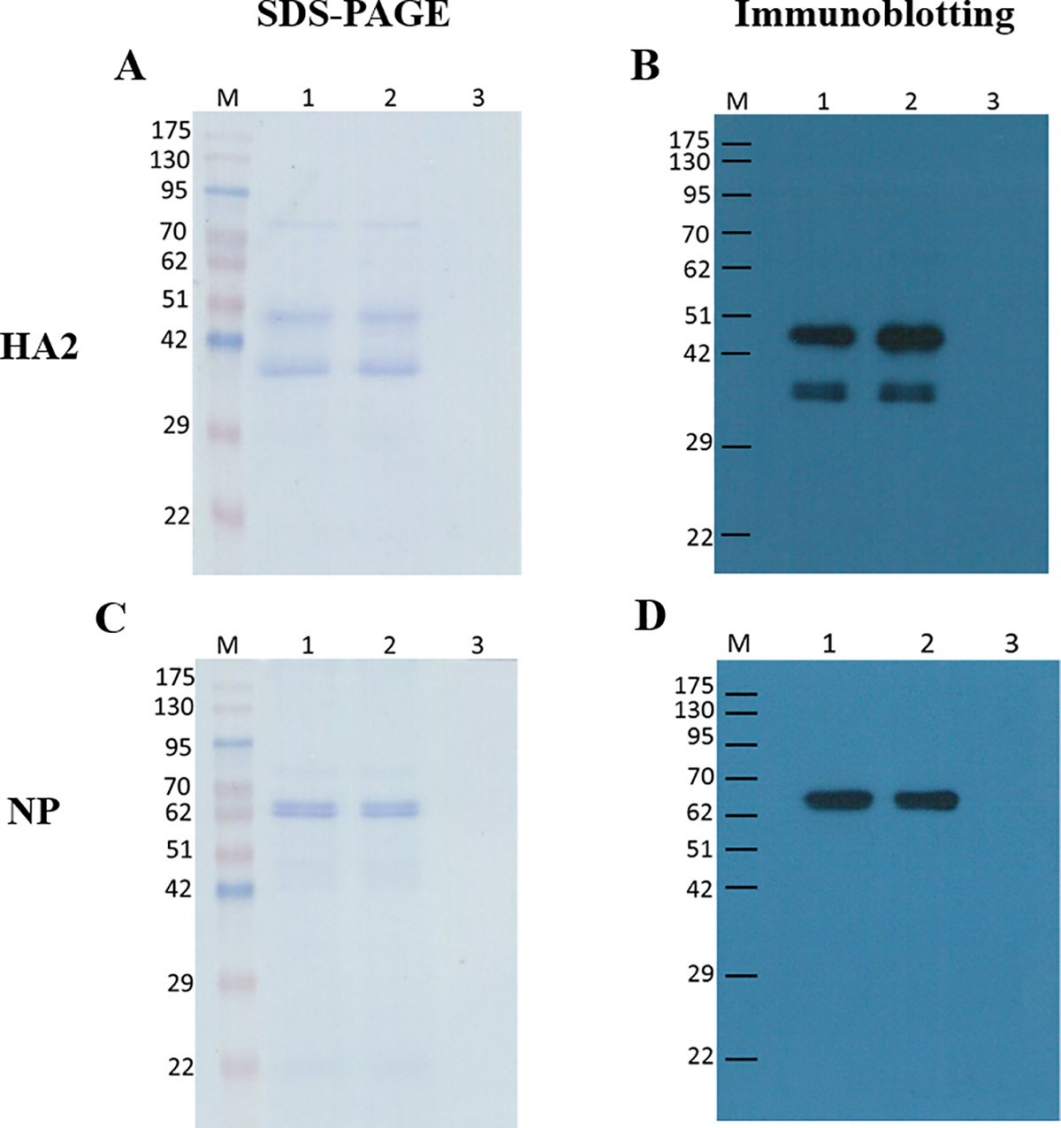
The stability of HA2 (A, B) and NP (C, D) in nanoparticles. SDS-PAGE (A and C) and immunoblotting using monoclonal antibodies against HA (B) or rabbit polyclonal antibodies against NP (D) after destabilization. HA2-TMC nPs and NP-TMC nPs were dissolved in 10% NaCl solution. The destabilized products were (A, B) M: molecular weight markers, lane1: free HA2 protein in 5 mM HEPES, pH 7.4, lane 2: HA2 extracted from the HA2-TMC nPs, and lane 3: empty TMC nPs. (C, D) M: molecular weight markers, lane1: free NP protein in 5 mM HEPES, pH 7.4, lane 2: NP extracted from the NP-TMC nPs, and lane 3: empty TMC nPs.

To detect whether the encapsulation process of HA2 and NP proteins into TMC nPs altered their antigenicity, protein-loaded TMC nPs were destabilized by adding NaCl, followed by SDS-PAGE and immunoblotting. Both free and entrapped antigen bands corresponding to HA2 (~34 and 44 kDa) and NP (~62 kDa) appeared similarly in SDS-PAGE. No additional bands indicating the presence of fragments and an irreversible aggregation were visible (Fig 1A and 1C). Immunoblotting analysis confirmed that the antigenicity of HA2 and NP was not altered following their entrapment into TMC nPs (Fig 1B and 1D). These results indicate that HA2 and NP proteins in TMC nPs were intact and their sizes and epitopes were preserved after encapsulation. Our TMC nPs preparation was suitable for entrapment of HA2 and NP proteins.

### Toxicity and cell viability

**Fig 2:**
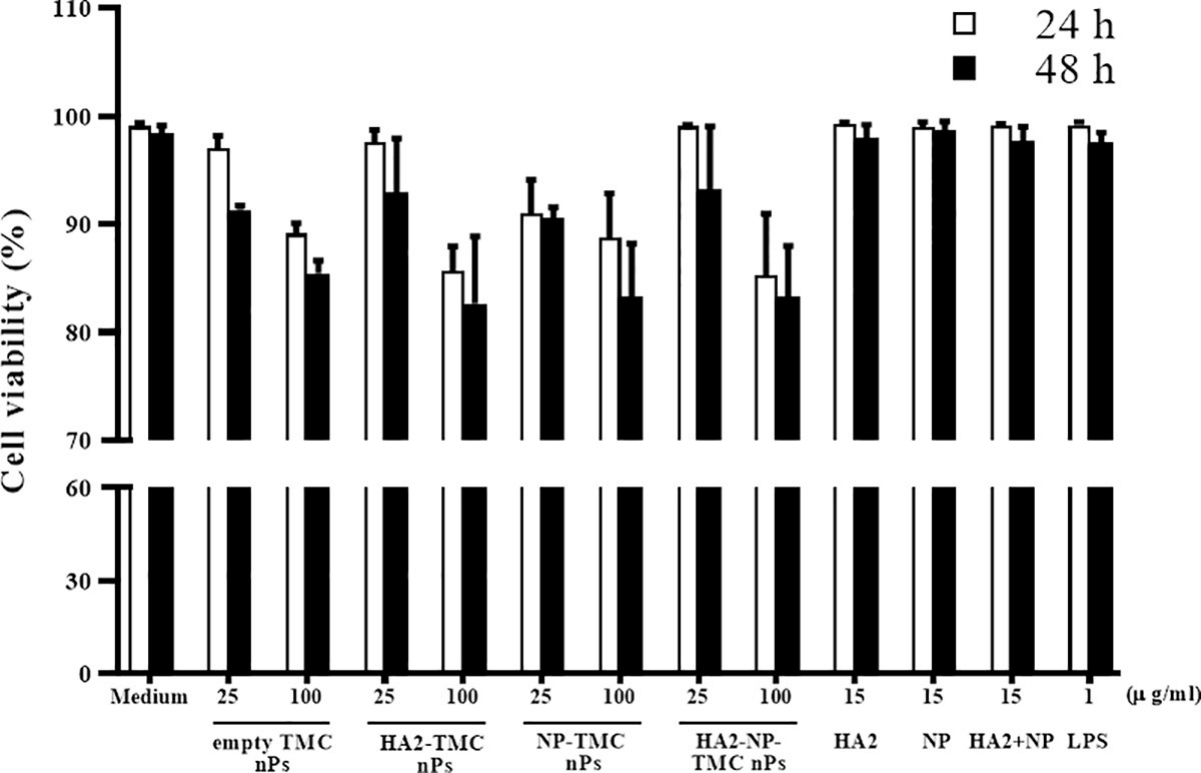
The toxicity of influenza nanoparticle constructs on cell viability. HNEpCs were treated with medium, LPS (1 μg/ml), protein alone (HA2, NP or HA2+NP at 15 μg/ml), empty TMC nPs, HA2-TMC nPs, NP-TMC nPs or HA2-NP-TMC nPs (25 and 100 μg/ml) for 48 h. Cell viability was quantitated by trypan blue exclusion.

To determine the effect of influenza nanoparticle constructs on cell viability, HNEpCs were treated with protein alone (HA2, NP or HA2+NP), empty TMC nPs, HA2-TMC nPs, NP-TMC nPs, HA2-NP-TMC nPs or LPS for 48 h (Fig 2). Cell viability was measured by trypan blue staining. Cell viability after empty TMC nPs treatment was 85.4 ± 1.21% at 100 μg/ml, after 48 h of treatment. Cell viability of HA2 or NP encapsulated into TMC nPs was similar to empty TMC nPs. Cell viabilities were 82.6 ± 6.2%, 83.3 ± 4.9%, and 83.2 ± 4.7%, at 100 μg/ml, after 48 h of treatment for HA2-TMC nPs, NP-TMC nPs and HA2-NP-TMC nPs, respectively. These results indicate that influenza nanoparticle constructs showed little toxicity in HNEpCs.

### Cellular uptake of nanoparticles

**Fig 3:**
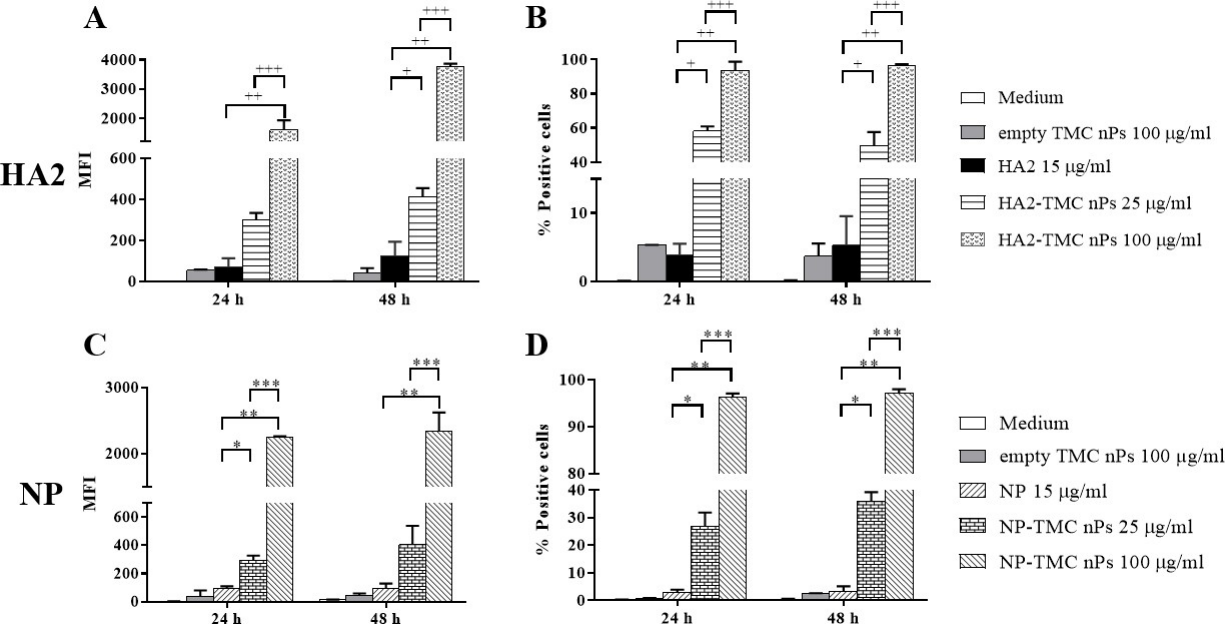
TMC nPs increase HA2 and NP antigen uptake by HNEpCs. Mean fluorescence intensity (MFI) and percentage of HA2 positive cells (A and B) and NP positive cells (C and D). HNEpCs were stimulated with medium, empty TMC nPs (100 μg/ml), protein alone (HA2 and NP at 15 μg/ml), HA2-TMC nPs and NP-TMC nPs at 25 and 100 μg/ml for 24 and 48 h and analyzed by flow cytometry. ^+^ and ^++^ denote significant difference between HA2 and HA2-TMC nPs at 25 or 100 μg/ml, respectively (*P* <0.05). ^+++^ denotes significant difference between HA2-TMC nPs at 25 and 100 μg/ml (*P* <0.05). * and ** denote significant difference between NP and NP-TMC nPs at 25 or 100 μg/ml, respectively (*P* <0.05). *** denotes significant difference between NP-TMC nPs at 25 and 100 μg/ml (*P* <0.05). Statistical significance was determined by one-way ANOVA with Tukey's post-test. The amounts of encapsulated HA2 or NP protein into TMC nPs at 25 and 100 μg/ml were 3.75 and 15 μg, respectively.

We used HNEpCs to investigate the cellular uptake of HA2 and NP proteins via TMC nPs. Treatment the cells with HA2-TMC nPs and NP-TMC nPs resulted in significantly (*P* <0.05) higher mean fluorescence intensity (MFI) and percentage of positive cells than those receiving HA2 and NP protein alone. HA2-TMC nPs (Fig 3A and 3B) and NP-TMC nPs (Fig 3C and 3D) at 100 μg/ml exerted the strongest MFI and the highest percentage of positive cells. In contrast, treatment with 15 μg/ml of HA2 or NP protein alone had the lowest. These results show that TMC nPs increase uptake of HA2 and NP proteins into HNEpCs in a dose- and time-dependent manner.

### Cytokine and chemokine productions by HNEpCs in response to nanoparticles

**Fig 4:**
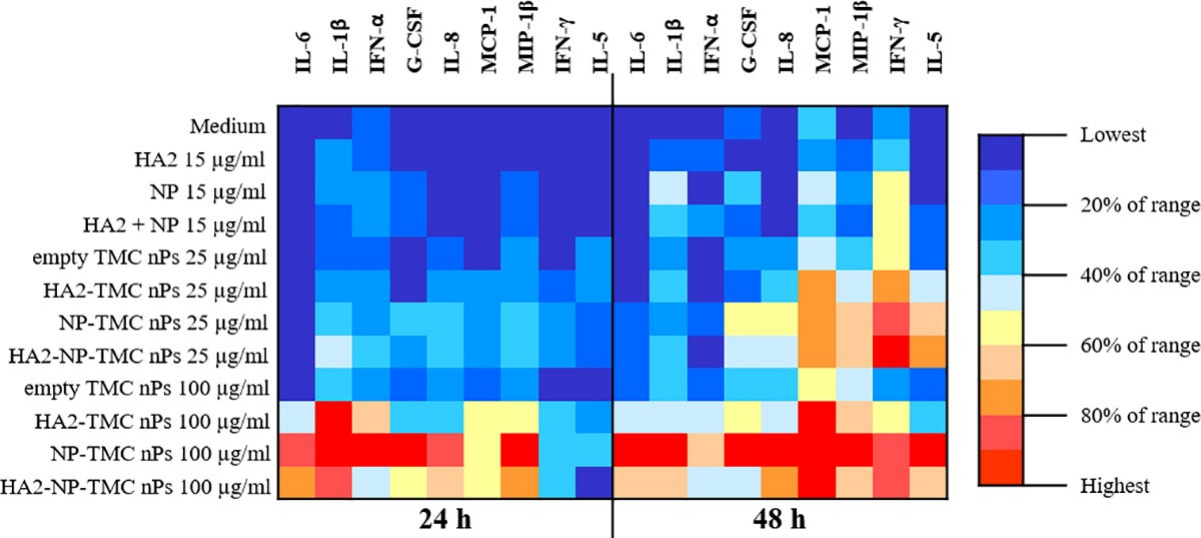
Heat map display of cytokine and chemokine productions. HNEpCs were treated with medium, protein alone (HA2, NP or HA2+NP at 15 μg/ml), empty TMC nPs, HA2-TMC nPs, NP-TMC nPs and HA2-NP-TMC nPs (25 and 100 μg/ml). Supernatant was collected after 24 and 48 h and cytokine and chemokine productions were measured by Bio-Plex bead-based assay. IFN-α was measured by IFN-α ELISA assay. The amounts of encapsulated HA2 or NP protein into TMC nPs were calculated at 25 and 100 μg/ml were 3.75 and 15 μg, respectively.

We investigated the immune stimulatory effects of influenza nanoparticle constructs by measuring cytokine and chemokine productions in the supernatants of HNEpCs treated with protein alone (HA2, NP or HA2+NP), empty TMC nPs, influenza nanoparticle constructs (HA2-TMC nPs, NP-TMC nPs, and HA2-NP-TMC nPs) or positive control (LPS). Nine cytokine and chemokine were detected in the supernatant and were categorized into pro-inflammatory cytokines (IL-6 and IL-1β), Type I interferon and growth factor (IFN-α and G-CSF), chemokines (IL-8, MCP-1 and MIP-1β) and, Th1-and Th2-related cytokines (IFN-γ and IL-5) (Fig 4 and S2 Fig). We found that soluble proteins (HA2, NP and HA2+NP) exerted low immune stimulation effect, compared to empty TMC nPs and influenza nanoparticle constructs. Pro-inflammatory cytokines (IL-6 and IL-1β) were induced at significantly higher levels (*P* <0.05) in HNEpCs-treated cells with influenza nanoparticle constructs as compared to cells treated with empty TMC nPs and protein alone. IL-6 and IL-1β were secreted in a dose-dependent manner. IFN-α secretion peaked at 24 h of treatment. Only HA2-TMC nPs and NP-TMC nPs at 100 μg/ml induced higher IFN-α production (*P* <0.05) compared to empty TMC nPs. All influenza nanoparticle constructs at 100 μg/ml induced higher G-CSF production than that of other groups at 24 h of treatment. Secretion of the chemokines IL-8, MCP-1 and MIP-1β followed a similar pattern of induction (*P* <0.05) when treated with all influenza nanoparticle constructs in dose- and time-dependent manners. Influenza nanoparticle constructs stimulated Th1-related IFN-γ production at 24 h of treatment and continuously increased at 48 h of treatment. At highest dose (100 μg/ml), all influenza nanoparticle constructs induce higher levels of secreted IFN-γ (*P* <0.05) compared with empty TMC nPs. Only NP-TMC nPs at 100 μg/ml induced significantly higher level (*P* <0.05) of IL-5 (Th2-related cytokine) production than empty TMC nPs at 48 h of treatment. Nine other cytokines, TNF-α, GM-CSF, IL-7, IL-2, IL-12p70, IL-17, IL-4, IL-10, and IL-13, could not be detected in the supernatants of HNEpCs.

### Influenza nanoparticle construct-treated HNEpCs generate soluble factors that induce MoDCs maturation

**Fig 5:**
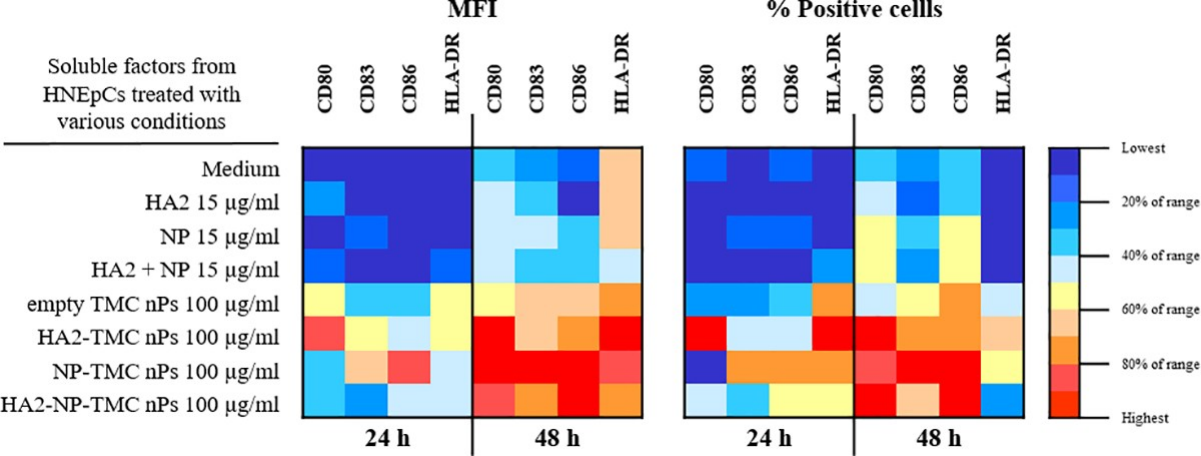
Soluble factors secreted by influenza nanoparticle construct-treated HNEpCs induce MoDCs maturation. HNEpCs were treated with medium, protein alone (HA2, NP or HA2+NP at 15 μg/ml), empty TMC nPs, HA2-TMC nPs, NP-TMC nPs and HA2-NP-TMC nPs (100 μg/ml). The supernatants of HNEpCs were collected at 48 h and added to MoDCs to determine the effect on MoDCs maturation. The expression levels of CD80, CD83, CD86, and HLA-DR was determined as mean fluorescence intensity (MFI) and percentage of positive cells by flow cytometry at 24 ad 48 h. The amount of encapsulated HA2 or NP protein into TMC nPs was calculated at 100 μg/ml was 15 μg.

The use of a TMC nPs as a delivery system may increase antigen uptake into human nasal epithelium which may subsequently activate dendritic cells, important mediators of inflammation. We investigated whether soluble factors secreted by nanoparticles-treated HNEpCs cells could drive MoDCs maturation. Soluble factors secreted by empty TMC nPs-treated HNEpCs upregulated expression of maturation markers (CD80, CD83, CD86 and HLA-DR) in MoDCs (Fig 5 and S3 Fig). In all influenza nanoparticle construct-treated conditions (HA2-TMC nPs, NP-TMC nPs, and HA2-NP-TMC nPs), CD80 expression could be detected at 24 h and was significantly upregulated (*P* <0.05) at 48 h of treatment compared with all soluble protein conditions (HA2, NP and HA2+NP). Surface expression of CD83, CD86 and HLA-DR in all influenza nanoparticle construct-treated conditions were significantly higher (*P* <0.05) than that of all soluble protein conditions at both time points. Soluble protein induced expression of markers was similar to MoDCs treated with medium alone. This may be due to the poor uptake of all proteins by HNEpCs (as shown in Fig 3) resulting in low cytokine and chemokine secretions. These data indicate that soluble factors secreted by influenza nanoparticle construct-treated HNEpCs were able to induce MoDCs maturation and cell surface expression of CD80, CD83, CD86 and HLA-DR markers.

### Efficacy of influenza nanoparticle construct-treated HNEpCs against influenza virus challenge

**Fig 6:**
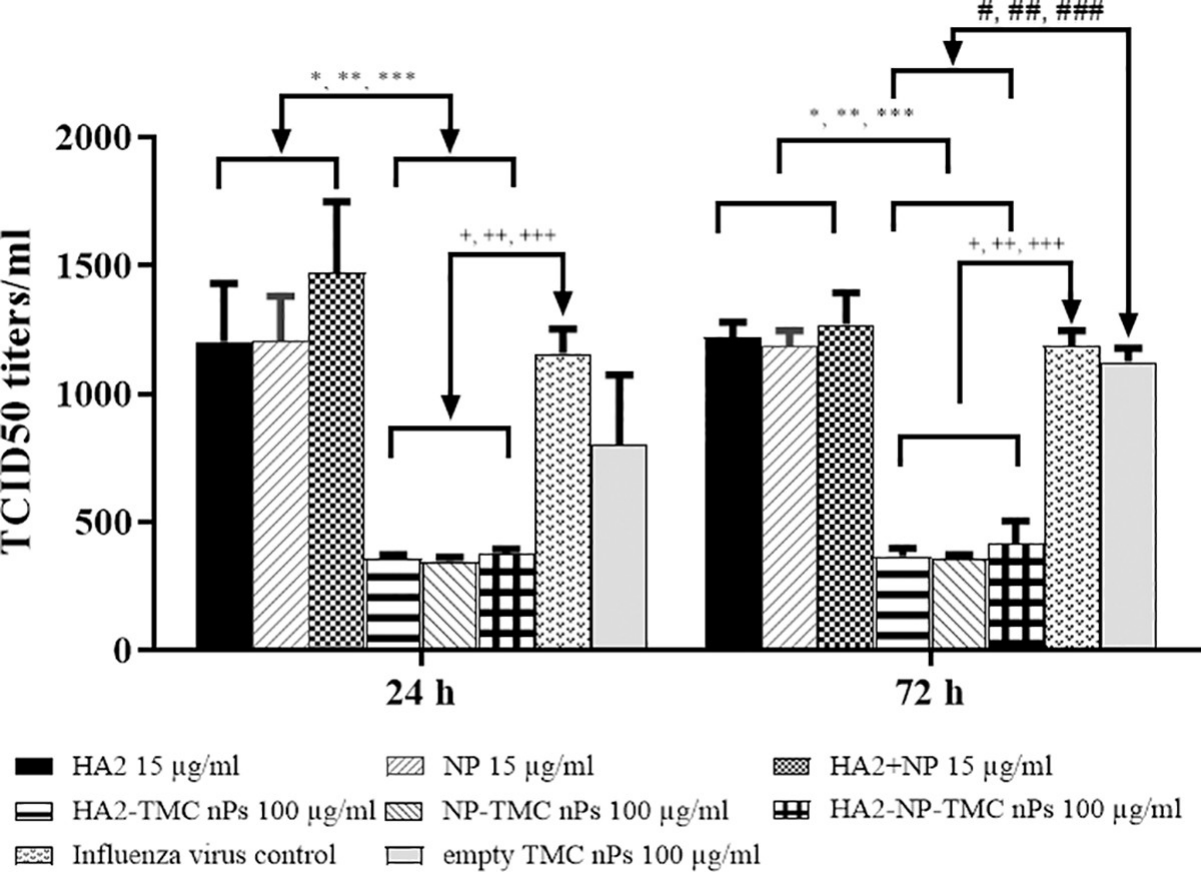
Influenza nanoparticle constructs inhibit influenza viral replication in HNEpCs. HNEpCs were pre-treated with protein alone (HA2, NP and HA2+NP at 15 μg/ml), influenza nanoparticle constructs (HA2-TMC nPs, NP-TMC nPs and HA2-NP-TMC nPs at 100 μg/ml) and medium alone for 24 h before being challenged with A/California/07/2009 (H1N1) at an MOI of 1.5. Supernatant was collected after 24 and 72 h and the amount of influenza virus replication was quantified by tissue culture infectious dose (TCID_50_). *, **, *** denotes significant difference between soluble protein and influenza nanoparticle constructs (*P* <0.05) (HA2 vs HA2-TMC nPs, NP vs NP-TMC nPs and HA2+NP vs HA2-NP-TMC nPs), respectively. ^+, ++, +++^ denotes significant difference between influenza nanoparticle constructs (HA2-TMC nPs, NP-TMC nPs and HA2-NP-TMC nPs) and influenza virus control (*P* <0.05), respectively. ^#, ##, ###^ denotes significant difference between influenza nanoparticle constructs (HA2-TMC nPs, NP-TMC nPs and HA2-NP-TMC nPs) and empty TMC nPs (*P* <0.05), respectively. One way ANOVA with Tukey's post-test was determined. The amounts of encapsulated HA2 or NP proteins into TMC nPs at 100 μg/ml were 15 μg.

To determine the efficacy of influenza nanoparticle construct against influenza virus propagation, HNEpCs were treated with the influenza nanoparticle constructs before challenging with A/California/07/2009 (H1N1) at an MOI of 1.5. The production of infectious viruses was quantitated using 50% tissue culture infectious dose (TCID_50_) assay. As revealed in Fig 6, all influenza nanoparticle construct-treated conditions (HA2-TMC nPs, NP-TMC nPs, and HA2-NP-TMC nPs) significantly downregulated (*P* <0.05) influenza virus productions compared with influenza virus control by approximately 65% to 70% at all time points. There was no significance difference between the inhibitory activity induced by soluble protein (HA2, NP and HA2+NP) or empty TMC nPs. These results indicate that influenza nanoparticle constructs inhibit influenza virus replication.

## Discussion

We developed novel influenza nanoparticle constructs composed of influenza HA2 or NP antigen, encapsulated into TMC nPs and tested their immunological and inhibitory properties in an *in vitro* system. These results demonstrate that TMC nPs are not only an efficient delivery system for nasal vaccines but also an adjuvant.

The HA2 and NP proteins were chosen because they contain highly conserved regions, which could deliver cross-protective immune responses against different subtypes of influenza A viruses. HA2 vaccines induce antibodies of broad neutralizing activity [33, 52, 53], targeting the HA2 subunit and preventing fusion during viral entry. NP vaccines have been shown to induce efficacious cytotoxic T cell responses [45]. *In vivo* models revealed that administration of recombinant NP induces protective immunity [41–46]. Given their highly conserved sequences, a universal influenza vaccine composed of HA2 and NP could provide cross protection against heterosubtypes of influenza viruses [47–49].

In this study, we use *P*. *pastoris* as an expression host for recombinant production of H1N1 HA2 and NP proteins. Our HA2 and NP proteins expressed in yeast were bigger in size than HA2 and NP proteins expressed in prokaryotic systems [52, 54, 55]. It is possible that recombinant proteins expressed in *E*.*coli* may be unable to correctly fold and undergo post-translational modifications such as glycosylation. Two band of our purified HA2 protein (~34 and 44 kDa) were detected by SDS-PAGE and immunoblotting (Fig 1A and 1B). When expressed in *P*. *pastoris* as a soluble secreted protein, the HA0 protein (HA1 and HA2 subunits) of the novel H1N1 A/California/04/2009 showed HA2 to be about 40–45 KDa [56]. That size was close to our bigger protein (44 KDa). However, our purified HA2 protein was expressed intracellularly and not secreted as we expected. It may include the complete completed (44 KDa) form and an incomplete (34 KDa) one from the HA2 proteins. The incomplete, 34 KDa, form of HA2 proteins may be the consequence of incomplete post-translational modifications.

Several studies demonstrated that TMC nPs loaded with influenza antigens are capable of eliciting strong systemic as well as local antibody responses after intranasal administration [26]. In this study, we encapsulated the HA2 and NP proteins into TMC nPs, showing high loading efficiency. Influenza antigen-loaded TMC nPs retain their antigenicity and are taken up by the nasal epithelium cells. Size, and surface charge play roles in antigen uptake. However, the optimal size for nasal vaccine delivery vehicles is still controversial. Several groups have reported the particles sized less than 1 μm (nanoparticle) are taken up by nasal epithelia and nasopharyngeal-associated lymphoid tissue (NALT) more efficiently than larger particles [57–60]. Durrey *et al* showed that mucoadhesive particles (230–320 nm) could penetrate through the mucus layer while particles large than 2 μm were retained on the surface of mucosa [61]. A previous study showed that TMC nPs (350 nm) are suitable for *in vivo* uptake by the nasal epithelium and NALT cells, and transported to sub-mucosal layers [62]. The optimum particle size for DC uptake is 500 nm and below [63]. Consistently, higher macrophage uptake of smaller PLA particles (200–600 nm) in comparison to larger ones (2–8 μm) has been reported [64]. HA2-TMC nPs and NP-TMC nPs were at 422.9 and 489.8 nm in size, respectively.

We use HNEpCs as an *in vitro* model of human nasal epithelium cells for assessing the intranasal delivery of influenza HA2 and NP proteins. We found that TMC nPs enhance HA2 and NP antigen uptake by HNEpCs (as shown in Fig 3). As previous reported, TMC nPs display mucoadhesive properties in their prolongation of residence time at the absorption site and in a wide range of pH [25]. Consistently with the *in vivo* model, the addition of TMC nPs brings whole inactivated influenza virus in much closer contact with the epithelial surfaces than WIV alone [65]. In this study, we established HA2-TMC nPs and NP-TMC nPs with positively surface charge. The uptake of influenza nanoparticle constructs may be mediated through the positive charge of influenza nanoparticle constructs and the negative charge of cell membranes. Consistent with previous report, Nantachit *et al* showed that HNEpCs are permissive to envelop domain III of dengue virus 3 (EDIII-D3) via TMC nPs [66]. EDIII-D3 TMC nPs were found to stimulate a strong local innate antiviral response, offering an alternative vaccine approach for nasal dengue vaccines.

We found that soluble HA2 and NP protein alone exerted low immunogenicity while, when encapsulated into TMC nPs induce high levels of cytokine and chemokine secretion. We revealed that influenza nanoparticle constructs induced pro-inflammatory cytokines. IL-6 plays role in the inflammatory response to pathogens. We showed that influenza nanoparticle constructs induced IL-1β secretion, common in inflammasome activation. Inflammasome activation are bound to have important roles in the host defense against pathogenic microorganisms [67]. Previous studies have reported that inflammasome associated *in vivo* innate immune response to influenza virus infection through the sensing of viral RNA [68]. Moreover, inflammasome activation are required in the development of adaptive immune responses and for protective immunity against influenza virus challenge [69]. We also found IFN-α secretion upon treatment with HA2-TMC nPs, and NP-TMC nPs. IFN-α is the first line of defense against influenza virus infections and critical for prevention of severe influenza virus infection. Previous study showed that NS1 protein of influenza virus increase the ability in the Type I IFN antagonism to invade the IFN system [70]. G-CSF, a growth factor for neutrophils, induces expansion and enhancement of phagocytosis of the monocyte/macrophage system. Additionally, influenza nanoparticle constructs significantly upregulated the levels of chemokines (IL-8, MCP-1 and MIP-1β) as well as the Th1- and Th2- related cytokine productions (IFN-γ and IL-5). Chemokines promote chemotaxis on different cell types such as neutrophils, monocytes/macrophages and natural killer cells to the site of infection. IFN-γ is a representative Th1 cytokine associated with inhibition of influenza A virus proliferation. It is an important activator of macrophages and inducer of MHC II molecule expression whereas IL-5 stimulates proliferation, and differentiation of B cells and eosinophils and also increases immunoglobulin secretion, primarily IgA. Both are the major effectors of viral clearance in term of T cell proliferation and B cell differentiation. Combined, these findings indicated that our influenza nanoparticle constructs, at least an *in vitro* model, may have the capacity to trigger both innate and adaptive immune responses.

We demonstrated that soluble factors in HA2-TMC nPs, NP-TMC nPs and HA2-NP-TMC nPs-treated HNEpCs significantly upregulated maturation markers (CD80, CD83, CD86 and HLA-DR), in MoDCs. In this study, soluble factors secreted by influenza nanoparticle constructs-treated cells, include IL-6, IL-1β, IFN-α, G-CSF, IL-8, MCP-1, MIP-1β, IFN-γ, and IL-5. IL-6 and IL-1β are involved in the inflammatory response. Chemokines may recruit immune cells while IFN-α and IFN-γ stimulate signalling pathways, including activator of transcription proteins. Taken together, the presence of these immune mediators might recreate an inflammatory environment, and enhance the recruitment of different immune system cells that are consequently able to induce MoDCs maturation. Treatment with empty TMC nPs also up-regulated expression of maturation markers at significantly higher levels than medium-treated cells. Previous studies showed that TMC nPs were able to stimulate DC maturation [24, 71]. Accordingly, it might be possible that these act synergistically to induce MoDCs maturation. These data suggest a potential role for TMC nPs as an additive adjuvant that can improve the ability of HA2 and NP to induce DCs maturation. We were also able to determine that our influenza nanoparticle constructs are significant inhibitors of influenza virus replication, whereas soluble protein alone (HA2, NP and HA2+NP) and empty TMC nPs were not. Addition of TMC nPs as carrier system might improve the ability of HA2 and NP immunogen to initiate cellular processes crucial for activation of innate and adaptive immune responses.

The significance of TMC nPs in serving as an additive adjuvant *in vitro* model is demonstrated by its low toxicity, ability to protect HA2 and NP from protein degradation, increase the uptake of HA2 and NP in primary human nasal epithelium cells, ability to induce cytokine and chemokine productions, and improve the ability of HA2 and NP to drive MoDCs maturation and reduce the influenza virus replication.

## Conclusion

Influenza nanoparticle constructs composed of HA2 or NP antigens loaded into TMC nPs were developed and tested in an *in vitro* system. TMC nPs act as a delivery system in nasal epithelial cells and may be used for intranasal vaccination. We show a potential mucosal delivery system for influenza immunogens and found it to stimulate local innate antiviral responses and possibly leading to systemic adaptive immunity. Influenza antigen-loaded TMC nPs should be further investigated in animal models to confirm their protective activity against influenza virus infection.

## Supporting information

S1 Fig Size distribution of empty TMC nPs, HA2-TMC nPs and NP-TMC nPs by dynamic light scattering. (TIF)

S2 Fig Cytokine and chemokine productions by HNEpCs in the response to nanoparticles. The HNEpCs were treated with medium, protein alone (HA2, NP or HA2+NP at 15 μg/ml), empty TMC nPs, HA2-TMC nPs, NP-TMC nPs and HA2-NP-TMC nPs (25 and 100 μg/ml). Supernatant was collected for 24 and 48 h and used to measure cytokine and chemokine productions by Bio-Plex bead-based assay as well as IFN-α ELISA assay. All productions were categorized into pro-inflammatory cytokines (A), Type I interferon and growth factor (B), chemokines (C), Th1-and Th-2 related cytokines (D). ^+^, ^++^ and ^+++^ denote significant differences between 25 μg/ml of empty TMC nPs and HA2-TMC nPs or NP-TMC nPs or HA2-NP-TMC nPs, respectively (*P* <0.05). *, ** and *** denote significant differences between 100 μg/ml of empty TMC nPs and HA2-TMC nPs or NP-TMC nPs or HA2-NP-TMC nPs, respectively (*P* <0.05). Statistical significance was determined by one-way ANOVA with Tukey's post-test. The amounts of encapsulated HA2 or NP proteins into TMC nPs at 25 and 100 μg/ml were 3.75 and 15 μg, respectively.
(TIF)

S3 Fig Expression of DC maturation markers are upregulated in the presence of the soluble factors secreted by influenza nanoparticle construct-treated HNEpCs. HNEpCs were treated with medium, LPS (1 μg/ml), protein alone (HA2, NP or HA2+NP at 15 μg/ml), empty TMC nPs, HA2-TMC nPs, NP-TMC nPs and HA2-NP-TMC nPs (100 μg/ml). The supernatants of HNEpCs from various conditions were collected at 48 h and stimulated the MoDCs to determine the effect on MoDCs maturation. The expression levels of CD80 (A), CD83 (B), CD86 (C), and HLA-DR (D) on various regimens-treated MoDcs were determined as mean fluorescence intensity (MFI) and percentage of positive cells by flow cytometry at 24 and 48 h. ^a^ denotes significant difference in MFI level between soluble factors secreted by medium and empty TMC nPs (*P* <0.05). * denotes significant differences in MFI level between soluble factors secreted by HA2 and HA2-TMC nPs (*P* <0.05). ** denotes significant differences in MFI level between soluble factors secreted by NP and NP-TMC nPs (*P* <0.05). *** denotes significant differences in MFI level between soluble factors secreted by HA2+NP and HA2-NP-TMC nPs (*P* <0.05). Statistical significance was determined by student t-test. The amounts of encapsulated HA2 or NP proteins into TMC nPs at 100 μg/ml was 15 μg.
(TIF)

S1 Raw Images (PDF)
