# Supplementary figures and images for: Development of an adjuvanted nanoparticle vaccine against influenza virus, an in vitro study
Source: PLoS One. 2020 Aug 6;15(8):e0237218. doi: 10.1371/journal.pone.0237218 (PMC7410248; doi:10.1371/journal.pone.0237218)

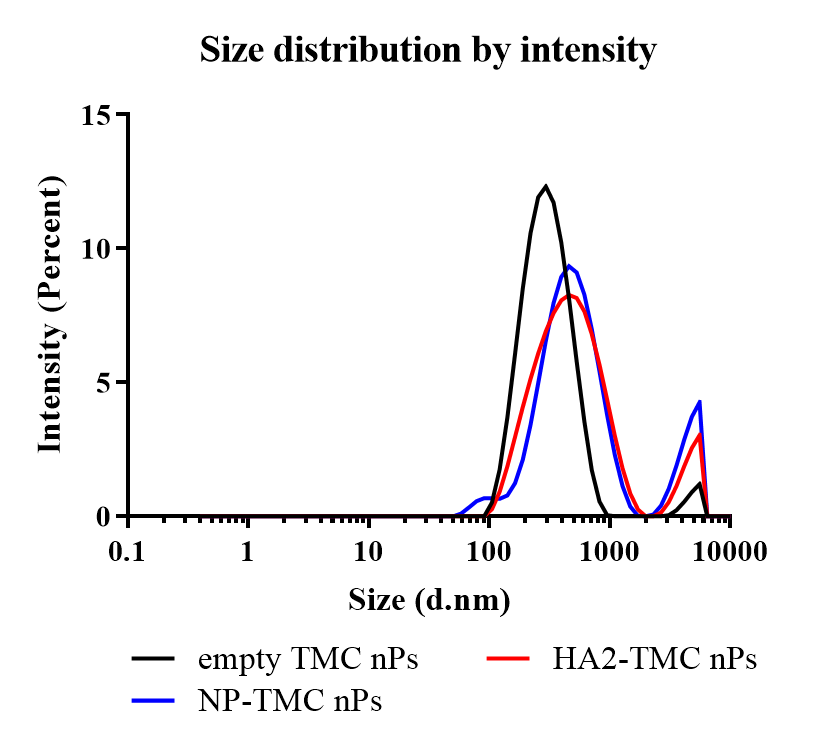

Supplement: S1 Fig — (TIF) [file pone.0237218.s001.tif]

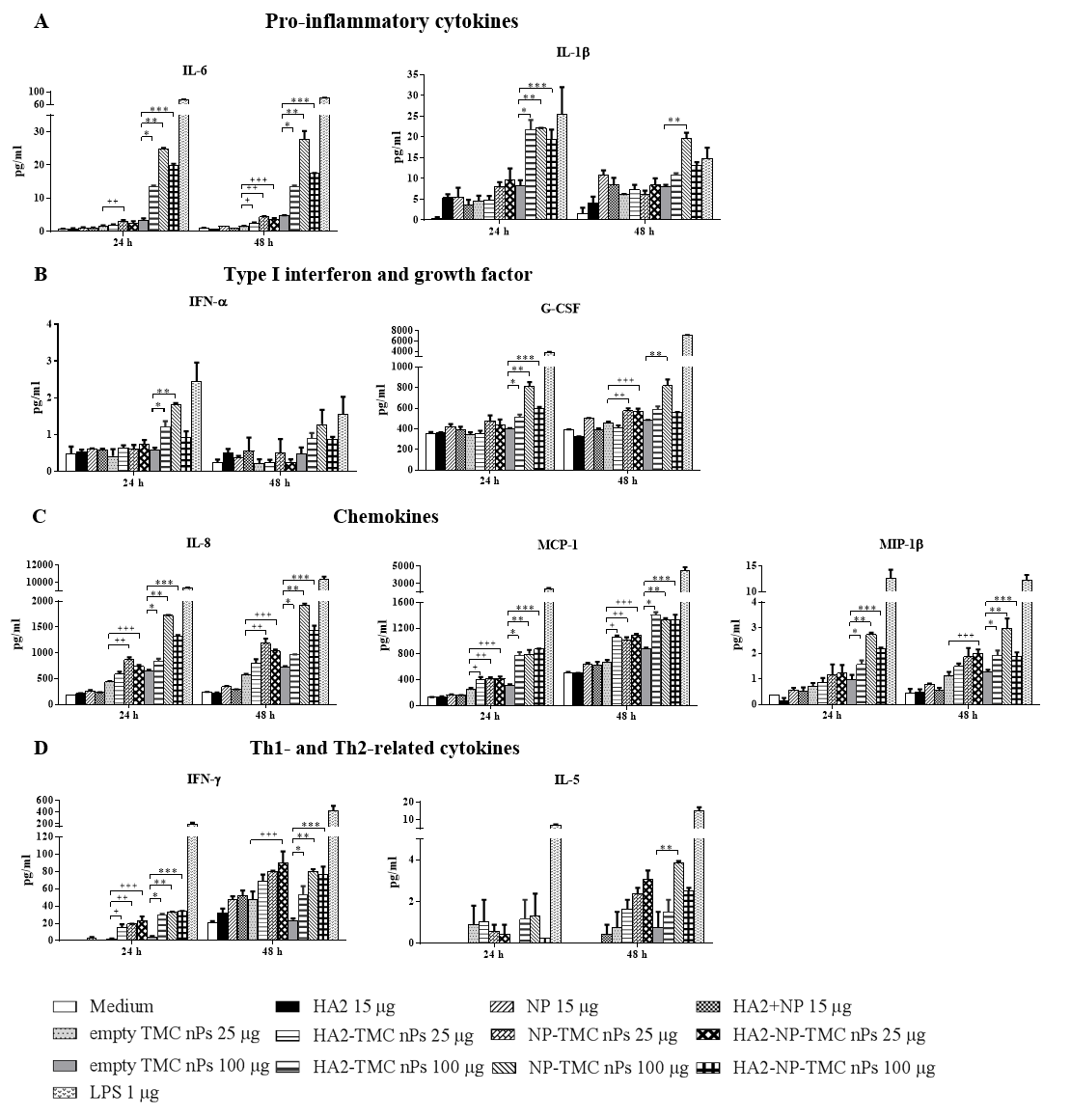

Supplement: S2 Fig — The HNEpCs were treated with medium, protein alone (HA2, NP or HA2+NP at 15 μg/ml), empty TMC nPs, HA2-TMC nPs, NP-TMC nPs and HA2-NP-TMC nPs (25 and 100 μg/ml). Supernatant was collected for 24 and 48 h and used to measure cytokine and chemokine productions by Bio-Plex bead-based assay as well as IFN-α ELISA assay. All productions were categorized into pro-inflammatory cytokines (A), Type I interferon and growth factor (B), chemokines (C), Th1-and Th-2 related cytokines (D). +, ++ and +++ denote significant differences between 25 μg/ml of empty TMC nPs and HA2-TMC nPs or NP-TMC nPs or HA2-NP-TMC nPs, respectively (P <0.05). *, ** and *** denote significant differences between 100 μg/ml of empty TMC nPs and HA2-TMC nPs or NP-TMC nPs or HA2-NP-TMC nPs, respectively (P <0.05). Statistical significance was determined by one-way ANOVA with Tukey's post-test. The amounts of encapsulated HA2 or NP proteins into TMC nPs at 25 and 100 μg/ml were 3.75 and 15 μg, respectively. (TIF) [file pone.0237218.s002.tif]

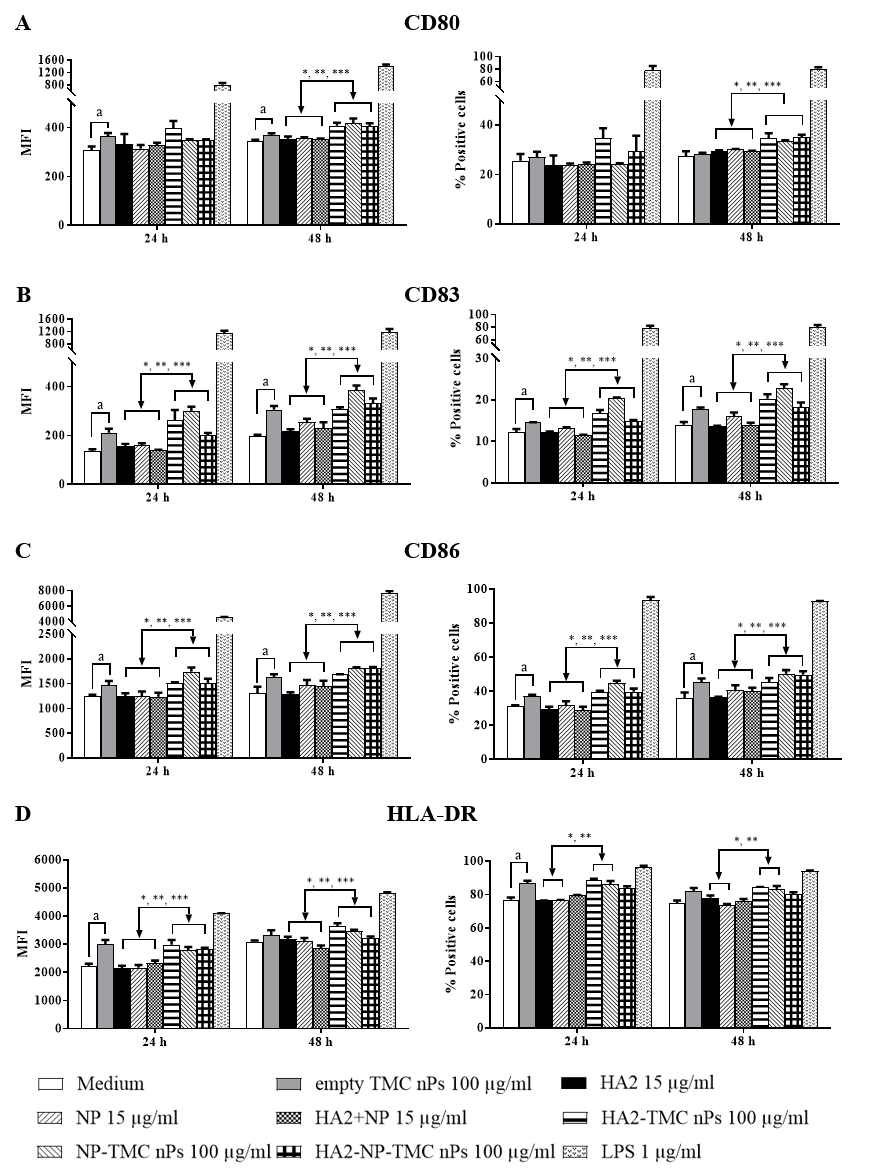

Supplement: S3 Fig — HNEpCs were treated with medium, LPS (1 μg/ml), protein alone (HA2, NP or HA2+NP at 15 μg/ml), empty TMC nPs, HA2-TMC nPs, NP-TMC nPs and HA2-NP-TMC nPs (100 μg/ml). The supernatants of HNEpCs from various conditions were collected at 48 h and stimulated the MoDCs to determine the effect on MoDCs maturation. The expression levels of CD80 (A), CD83 (B), CD86 (C), and HLA-DR (D) on various regimens-treated MoDcs were determined as mean fluorescence intensity (MFI) and percentage of positive cells by flow cytometry at 24 and 48 h. a denotes significant difference in MFI level between soluble factors secreted by medium and empty TMC nPs (P <0.05). * denotes significant differences in MFI level between soluble factors secreted by HA2 and HA2-TMC nPs (P <0.05). ** denotes significant differences in MFI level between soluble factors secreted by NP and NP-TMC nPs (P <0.05). *** denotes significant differences in MFI level between soluble factors secreted by HA2+NP and HA2-NP-TMC nPs (P <0.05). Statistical significance was determined by student t-test. The amounts of encapsulated HA2 or NP proteins into TMC nPs at 100 μg/ml was 15 μg. (TIF) [file pone.0237218.s003.tif]
