# Supplementary material for: Development of an adjuvanted nanoparticle vaccine against influenza virus, an in vitro study
Source: PLoS One. 2020 Aug 6;15(8):e0237218. doi: 10.1371/journal.pone.0237218 (PMC7410248; doi:10.1371/journal.pone.0237218)
Supplement: S1 Raw Images — (PDF) [file pone.0237218.s004.pdf]

<u>**Original images for blots and gels (Figure 1A-D)**</u>

1. **Figure panel from original images: SDS-PAGE, Figure 1A (Left) and 1C (Right)**

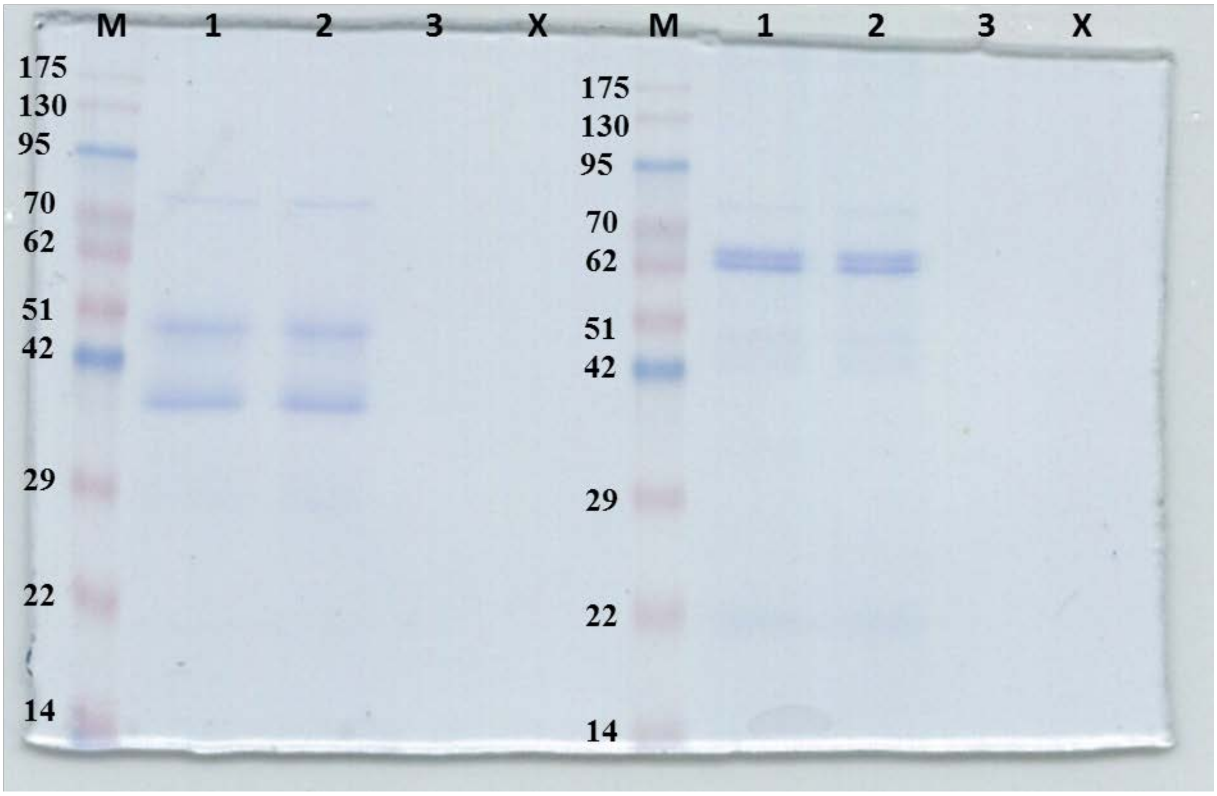

Fig 1A (Left): SDS-PAGE

-M: molecular weight markers, lane1: free HA2 protein in 5 mM HEPES, pH 7.4, lane 2: HA2 extracted from the HA2-TMC nPs, and lane 3: empty TMC nPs

-Use Fax scanner to capture this image

Fig 1C (Right): SDS-PAGE

-M: molecular weight markers, lane1: free NP protein in 5 mM HEPES, pH 7.4, lane 2: NP extracted from the NP-TMC nPs, and lane 3: empty TMC nPs

-Use Fax scanner to capture this image

2. **Figure panel from original images: Blots, Figure 1B (Left) and 1D (Right)**

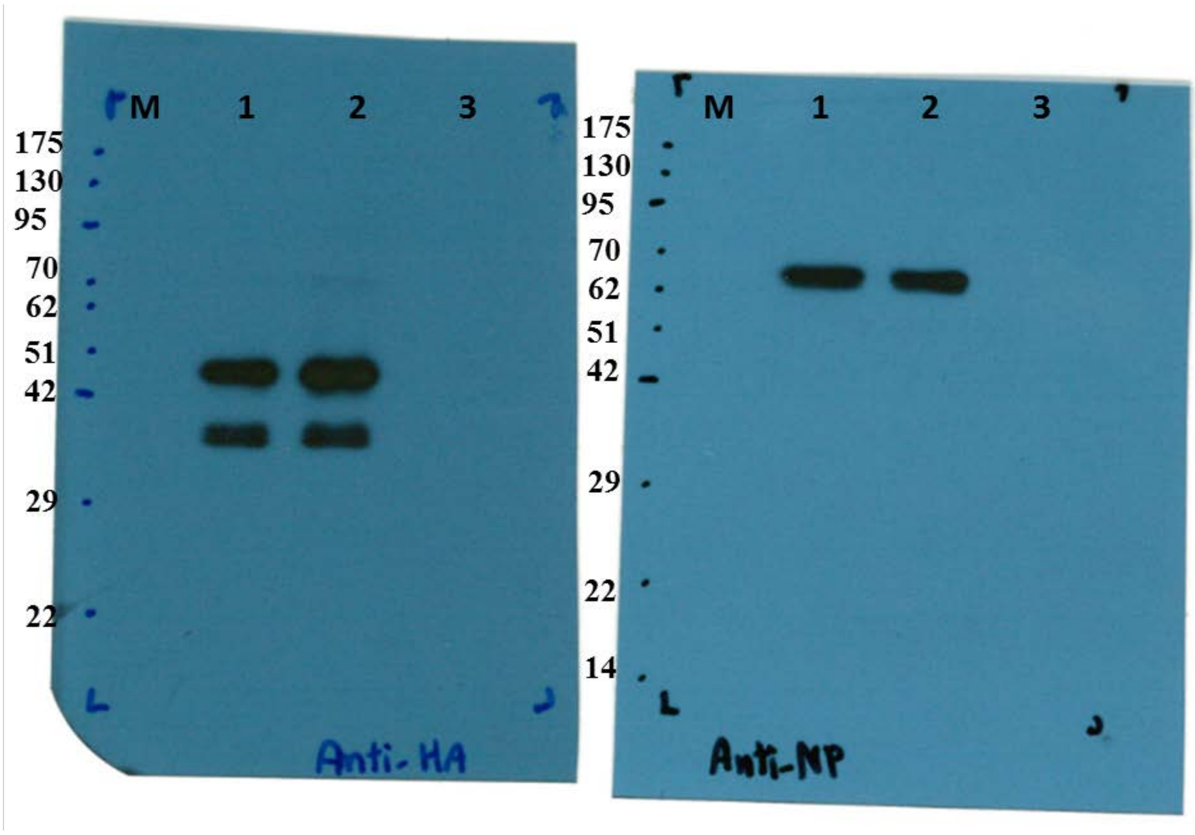

Fig 1B (Left): Blot using monoclonal antibodies against HA

-M: molecular weight markers, lane1: free HA2 protein in 5 mM HEPES, pH 7.4, lane 2: HA2 extracted from the HA2-TMC nPs, and lane 3: empty TMC nPs

-Use Fax scanner to capture this image

Fig 1D (Right): Blot using rabbit polyclonal antibodies against NP

-M: molecular weight markers, lane1: free NP protein in 5 mM HEPES, pH 7.4, lane 2: NP extracted from the NP-TMC nPs, and lane 3: empty TMC nPs

-Use Fax scanner to capture this image
